# Anti-tumor activity of BET inhibitors in androgen-receptor-expressing triple-negative breast cancer

**DOI:** 10.1038/s41598-019-49366-9

**Published:** 2019-09-16

**Authors:** In Hae Park, Han Na Yang, Su Yeon Jeon, Jung-Ah Hwang, Min Kyeong Kim, Sun-Young Kong, Sung Hoon Shim, Keun Seok Lee

**Affiliations:** 1 Translational Cancer Research Branch, Division of Cancer Research, National Cancer Center, Goyang, Korea; 2 Center for Breast Cancer, National Cancer Center, Goyang, Korea; 3 Genomic core, Omics Core Laboratory, National Cancer Center, Goyang, Korea; 4 Cancer Biomedical Science, National Cancer Center Graduate School of Cancer science and Policy, Goyang, Korea; 5 Department of Laboratory Medicine, Center for Diagnostic Oncology, National Cancer Center, Goyang, Korea

**Keywords:** Breast cancer, Targeted therapies

## Abstract

Triple-negative breast cancer (TNBC) is a heterogeneous disease comprising several subtypes. Androgen-receptor (AR) signaling has been targeted by several investigational agents in luminal AR subtype TNBCs. Bromodomain (BRD) and extra-terminal motif (BET) protein inhibitors have been shown to attenuate AR signaling in metastatic castration-resistant prostate cancer and to overcome enzalutamide resistance. We demonstrated potent anti-tumor effects of the BET inhibitor JQ1 against AR-positive TNBC cell lines using cell viability and cell cycle analysis. To reveal the mechanisms of JQ1 effects, multiplex gene expression analysis and immunoblotting assays were used. We examined *in vivo* effects of JQ1 in a xenograft model of AR expressing TNBC. JQ1 exhibited its anti-proliferative activity by inducing apoptosis and cell cycle arrest. JQ1 activity was not mediated by *MYC* downregulation. Instead, JQ1 blocked the interactions among the ATPase-family AAA-domain-containing 2 protein (ATAD2), BRD2, BRD4, and AR; effectively suppressing the expression of AR associated targets. In addition, JQ1 showed significant anti-tumor activity *in vivo* in TNBC xenograft mouse models as a monotherapy and in combination with anti-AR therapy. Taken together, our results showed that the BET inhibitor JQ1 is a promising therapeutic agent for the treatment of AR-positive TNBC.

## Introduction

Triple-negative breast cancer (TNBC) accounts for ~20% of metastatic breast cancer^1^. Cytotoxic chemotherapy is still known as the primary treatment modality for TNBC due to the lack of specific targets, including the estrogen receptor, progesterone receptor, and human epidermal growth factor 2 and its outcomes are poor compared to those of other breast cancer subtypes^2^. TNBC is a heterogeneous disease, encompassing distinct molecular subtypes^3–5^. The luminal androgen-receptor (LAR) subtype, accounting for ~20% of TNBCs, demonstrates increased expression of mRNA encoding the androgen receptor (AR), enhancing AR-signaling sensitive to AR antagonists^3^. AR expression in TNBC attenuates chemotherapy sensitivity and reduces the rates of complete remission after neoadjuvant treatment^6,7^. Several preclinical and early clinical studies suggest that AR is a potential therapeutic target in AR-positive (AR+) TNBC^8–10^. In a recent phase II clinical trial, anti-androgen therapy with bicalutamide or enzalutamide demonstrated relatively modest clinical efficacy in a selected group of patients, with a clinical benefit rate of 19 to 29% at 24 weeks^11,12^.

Considering the complexity of AR signaling and crosstalk in AR + breast cancer, AR antagonists have been investigated in combination with other targeted therapies. Such studies have focused on elucidating the mechanisms of primary and secondary resistance to AR inhibitors and on improving the efficacy of AR inhibitors in AR + breast cancers^13^. Actually, the mechanisms of resistance to AR blockade have been extensively characterized in prostate cancer. Acquired resistance to AR blockade comes from in various ways including AR overexpression, copy number amplification, as well as the expression of splice variants of AR to sustain AR activity^14^.

Bromodomain (BRD) and extra terminal domain (BET) family proteins control transcriptional activities by modulating protein assembly, and protein-protein interactions, contributing to a variety of cellular events, such as cell cycles, proliferation, and differentiation^15^. The BET family consists of four members, including BRD-containing protein 2 (BRD2), BRD3, BRD4, and BRD testis-specific protein BRDT^16^. BET inhibitors exhibit anti-tumor activity in various types of malignancies by inhibiting BRD4 and suppressing transcription of the *MYC* oncogene^16–19^. In addition, BRD4 interacts with the N-terminal domain of AR and induces AR-mediated gene transcription and BET inhibitor, JQ1 effectively disrupts the interaction between BRD4 and AR leading to cytotoxic efficacies in advanced prostate cancer model^17^. Recently, androgen regulated BRDs such as BRD2 and ATAD2 increased chromatin accessibility with enhancing AR recruitment to chromatin, which may drive cancer progression^20^. Given that BET family proteins interact with AR, BET inhibitors could be an alternative strategy for targeting AR-driven cancers. Consequently, we assessed the ability of the BET inhibitor JQ1 to enhance the efficacy of AR blockade in AR-expressing TNBCs.

## Materials and Methods

### Cell culture

Breast cancer cell lines, purchased from ATCC, were cultured according to supplier protocols. MDA-MB-231 cells were cultured in Roswell Park Memorial Institute (RPMI) 1640 medium. MDA-MB-453 and MDA-MB-468 cells were cultured in Leibovitz’s L-15 medium. BT-20 cells were cultured in Eagle’s Minimum Essential Medium. All cultures were supplemented with 1% antibiotics (Amphotericin B, Penicillin, Streptomycin) and 10% fetal bovine serum (GIBCO BRL). MDA-MB-231 and BT-20 cells were cultured at 37 °C in a 5% CO_2_ atmosphere. All other cell lines were cultured at 37 °C in free gas exchange with atmospheric air.

### Cell viability and cell cycle analysis

For cell viability measurement, the breast cancer cells were treated with 0.1–50 μM (+)-JQ1 and enzalutamide (Selleck Chemicals) diluted in dimethyl sulfoxide (DMSO). Cells were seeded in 96-well culture plates 24 hours prior to drug treatment, followed by exposure to indicated concentrations of compounds for 0–72 hours. 10 nM 5α-Dihydrotestosterone (DHT; Sigma-Aldrich, MO, USA) was added to charcoal-stripped serum media (Biowest, MO, USA) to evaluate drug effects on DHT-mediated proliferation of breast cancer cell lines. Cell viability was measured using the Cell Counting Kit-8 (Dojindo Molecular Technologies, MD, USA). For cell cycle analysis, drug-treated cells were stained with propidium iodide for quantitative analysis of DNA content. Analysis of the stained cells was performed using a BD LSRFortessa™ flow cytometer (BD bioscience, USA). Data was analyzed using FlowJo 10 Software (TreeStar Inc., USA).

### Small interfering (si)RNA

AR signaling in MDA-MB-231 and MDA-MB-453 cells was inhibited using siRNA silencing of genes encoding AR (Bioneer, Korea, #1008283, #1008282, #1008273), BRD2 (#1013117, #1013119, #1013121), BRD4 (#1013146, #1013144, #1013148), ATPase family, AAA domain containing 2 (ATAD2 #1009328, #1009324, #1009327), and MYC (#1100224, #1100229, #1100225). Cells were transfected with 10 pmol of indicated siRNA or of negative control siRNA using Lipofectamine® RNAiMAX (Invitrogen) according to manufacturer’s protocols. Briefly, cells were treated with the siRNA complex and incubated at 37 °C and 5% CO_2_ for 24–72 hours. After 6 hours, the complex-containing medium was removed and replaced with fresh medium. Transfection efficiency was determined using relative RNA and protein levels of each siRNA target in the transfected cells.

### Nuclear–cytoplasmic fractionation

A total of 1 × 10^6^ cells were seeded in 10-cm dishes in medium supplemented with 5% charcoal-stripped serum (Biowest, MO, USA) and incubated for 72 hours. Then, cells were pretreated with or without 30 uM enzalutamide for 3 hours followed by co-treatement with DHT for 3 hours. After that, cells were washed with PBS and cellular fractionation was performed using the NE-PER Nuclear and Cytoplasmic Extraction Kit (Thermo Fisher Scientific, MA, USA) as per manufacturer’s instructions. Protein of the isolated nucleus and cytoplasm was separated on SDS-PAGE and transferred to PVDF membrane. Immunoblot was performed using rabbit-anti AR, TOPO-I, a-tubulin and HRP-conjugated secondary antibodies.

### RNA extraction and quantitative (q)PCR

Total RNA was extracted from the breast cancer cells using the RNeasy Mini Kit (QIAGEN, Hilden, Germany). Genomic DNA was removed using DNase I (QIAGEN, Hilden, Germany). Synthesis of cDNA was performed using 500 ng of total RNA and the SuperScript ® III First-Strand Synthesis System (Invitrogen, CA, USA) at 50 °C for 50 min. Quantitative (qPCR) was performed using a LightCycler® 96 Real-Time PCR System (Roche Diagnostics, Germany). Relative mRNA expression was quantified using the LightCycler® 96 software (Roche Diagnostics) and normalized to ß-actin transcript levels. The following qPCR conditions were used: 45 cycles of 95 °C for 15 sec and 60 °C for 60 sec.

### Multiplex gene expression analysis

To identify cell signaling pathways affected by JQ1, multiplex gene expression analysis was performed using the nCounter® PanCancer Pathways Panel. Following JQ1 treatment, RNA was extracted from MDA-MB-231 and MDA-MB-453 cells as described above. The extracted RNA was subjected to an nCounter® PanCancer Pathways Panel (NanoString Technology, WA, USA) according to the manufacturer’s protocol. Briefly, capture probes, reporter probes, and total RNA were mixed with hybridization buffer and hybridized at 65 °C overnight. Then, the samples were processed on the nCounter Prep Station with wash reagents and an imaging cartridge. Hybridization occurred over 20 hours and was monitored in selected experiments.

### Antibodies, immunoblotting, and co-immunoprecipitation analyses

Mouse IgG against AR, BRD2, BRD4, ATAD2, and MYC (Abcam Cambridge, UK) were used for immunoprecipitation assays, and rabbit IgG against AR, BRD2, BRD4, ATAD2, MYC, poly (ADP-ribose) polymerase (PARP), cappase-9, caspase-3, and β-actin (CST, MA, USA) were used for immunoblotting. All antibodies were used at dilutions suggested by the manufacturers. For Western blot analysis, 50 μg of total protein extract was separated by SDS-PAGE and transferred to aNovex™ polyvinylidene fluoride (PVDF) membrane (Thermo Fisher Scientific, MA, USA) using an iBlot2™ Gel Transfer Device (Thermo Fisher Scientific, MA, USA). The blots were incubated overnight at 4 °C with primary antibodies diluted 1:500 or 1:1,000, and binding was detected using horseradish peroxidase (HRP)-conjugated secondary antibodies (1:2000 or 1:4000; Bio-Rad Laboratories, USA). Binding was detected using X-ray film via enhanced chemiluminescence with an Amersham ECL Western Blotting Detection Reagent (GE Healthcare, IL, USA).

For co-immunoprecipitation experiments, protein extracts (0.1 mg) were incubated overnight at 4 °C with antibodies against AR, BRD2, BRD4, ATAD2, or MYC or with mouse IgG antibody. The antibodies and bound proteins were purified using Protein A/G Sepharose beads (GE Healthcare, IL, USA). The protein complexes were washed 3 times at 4 °C with lysis buffer and subsequently subjected to SDS-PAGE under reducing conditions and immunoblotting as described above.

### *In vivo* tumor xenograft model

Female BALB/c nude mice, aged 5–6 weeks, were purchased from ORIENT BIO Inc. MDA-MB-231 cells were collected, washed with phosphate-buffered saline (PBS), and re-suspended in serum-free medium at a concentration of 1 × 10^7^ cells. Tumor cells resuspended in a Matrigel^®^ matrix (BD, NJ, USA) were implanted subcutaneously on the dorsal hind flank of 6–7 week-old BALB/c nude mice. Tumor size was measured every 3 days using an *in vivo* imaging system or a caliper, and drug treatment was initiated when tumors reached 100 mm^3^ in volume. The mice were divided into the following randomized groups: JQ1 treatment group (50 mg/kg, n = 4), enzalutamide treatment group (30 mg/kg, n = 4), combination group (50 mg/kg JQ1 and 30 mg/kg enzalutamide, n = 4), and vehicle group (control, n = 4). Mice were administered JQ1 (5% DMSO in 5% dextrose) and/or enzalutamide (2% DMSO in 30% PEG 300, 5% Tween 80) daily via intraperitoneal injection and oral gavage for 3 weeks. This study was reviewed and approved by the Institutional Animal Care and Use Committee (IACUC) of the National Cancer Center Research Institute, an AAALAC International-accredited facility that abides by the Institute of Laboratory Animal Resources guidelines.

### Immunohistochemical staining

Indicated TNBC cells were stained using an anti-AR antibody (Abcam Cambridge, UK) and 4′,6-diamidino-2-phenylindole (DAPI, Sigma-Aldrich corp., MO, USA). The cells were washed with PBS, fixed for 5 min with 4% paraformaldehyde at room temperature, washed again with PBS, permeabilized with 0.1% Triton X-100 in PBS for 10 min, and then blocked with 3% normal goat serum in PBS for 10 min. Following overnight incubation at 4 °C with the primary antibody diluted 1:200, the cells were incubated for 60 min with rabbit-Alexa Fluor 488 conjugated secondary antibody (1:400; Invitrogen^TM^), according to the manufacturer’s protocol. The staining was visualized using a Zeiss LSM780 confocal Microscope System (Carl Zeiss, Inc., Oberkochen, Germmany).

### Statistical analyses

Statistical significance was determined via the Mann-Whitney U test, Student’s *t*-test or ANOVA using the GraphPad Prism software.

### Ethics approval and consent to participate

This study was reviewed and approved by the Institutional Animal Care and Use Committee (IACUC) of the National Cancer Center Research Institute, an AAALAC International-accredited facility that abides by the Institute of Laboratory Animal Resources guidelines.

## Results

### AR inhibition modestly suppressed proliferation of AR + TNBC cells

**Figure 1:**
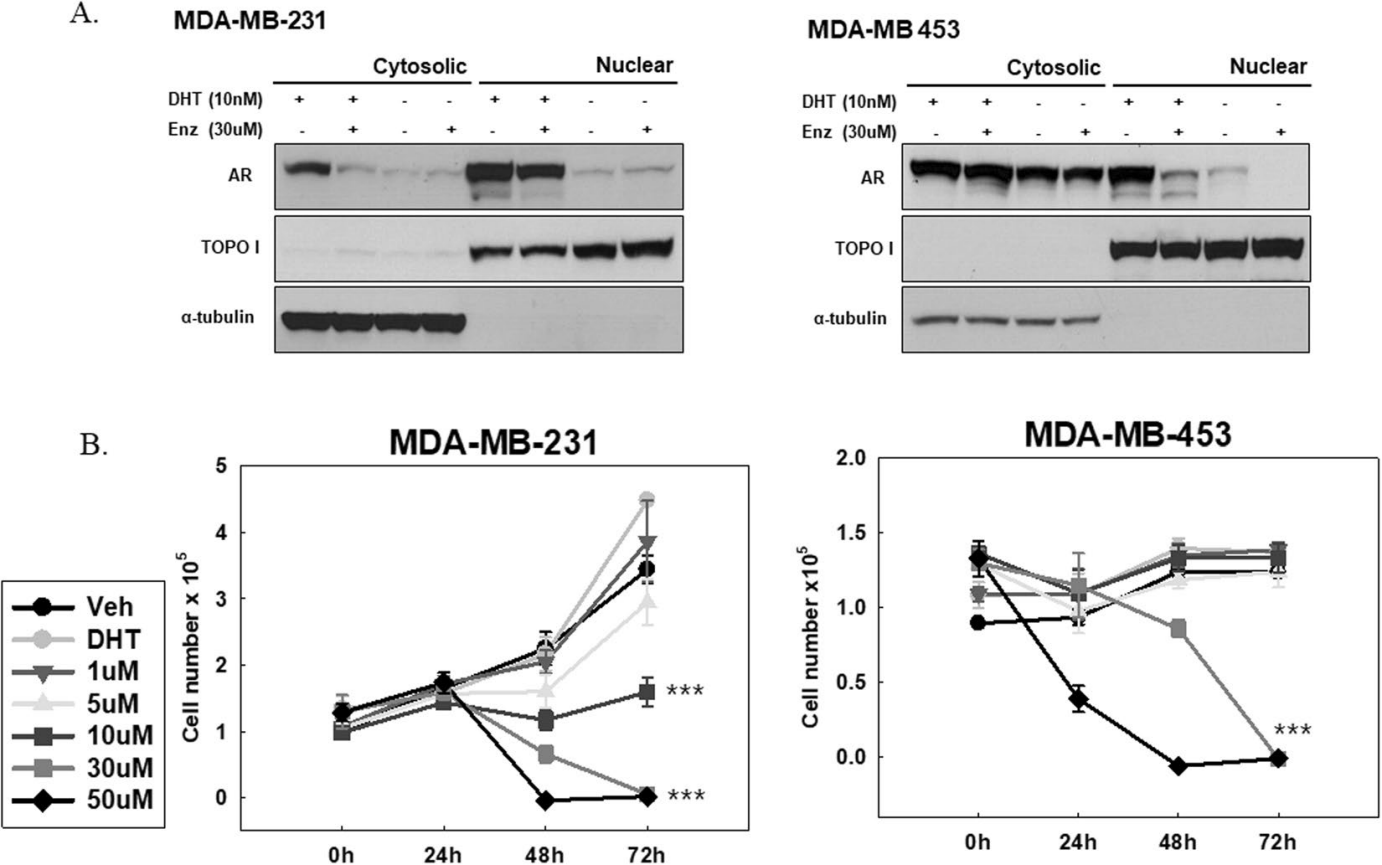
Enzalutamide (Enz) treatment of MDA-MB-231 and MDA-MB-453 cells in charcoal-stripped serum media with or without 10 nM 5-alpha-dihydrotestosterone (DHT) for 72 hours. (**A**) AR nuclear localization was blocked by 30 uM enzalutamide treatment in both cell lines. (**B)** Proliferation assays were used to determine the effect of enzalutamide on both cell lines. Error bars represent standard error of the mean (SEM). ***P ≤ 0.001, as determined using a two-tailed Student’s *t*-test. T- test was performed to compare with control at 72 hours.

To explore the correlation between enzalutamide sensitivity and AR expression, we evaluated AR transcript and protein abundance in selected TNBC cell lines (Supplemental Fig. 1a–c). The LAR subtype MDA-MB-453 and mesenchymal subtype MDA-MB-231 cells exhibited the two highest levels of AR mRNA and protein production. When AR inhibition was assessed in the AR + TNBC cell lines in charcoal-stripped serum media with or without 10 nM DHT, enzalutamide significantly blocked AR nuclear localization in both AR- expressing cell lines (Fig. 1A). However, the proliferation-suppressing effect of enzalutamide was moderate, and DHT treatment did not increase baseline proliferation in AR + TNBC cell lines (Fig. 1B). As expected, cell lines not expressing AR did not respond to enzalutamide treatment (Supplemental Fig. 2).

### BET inhibition induced apoptosis in TNBC cell lines

**Figure 2:**
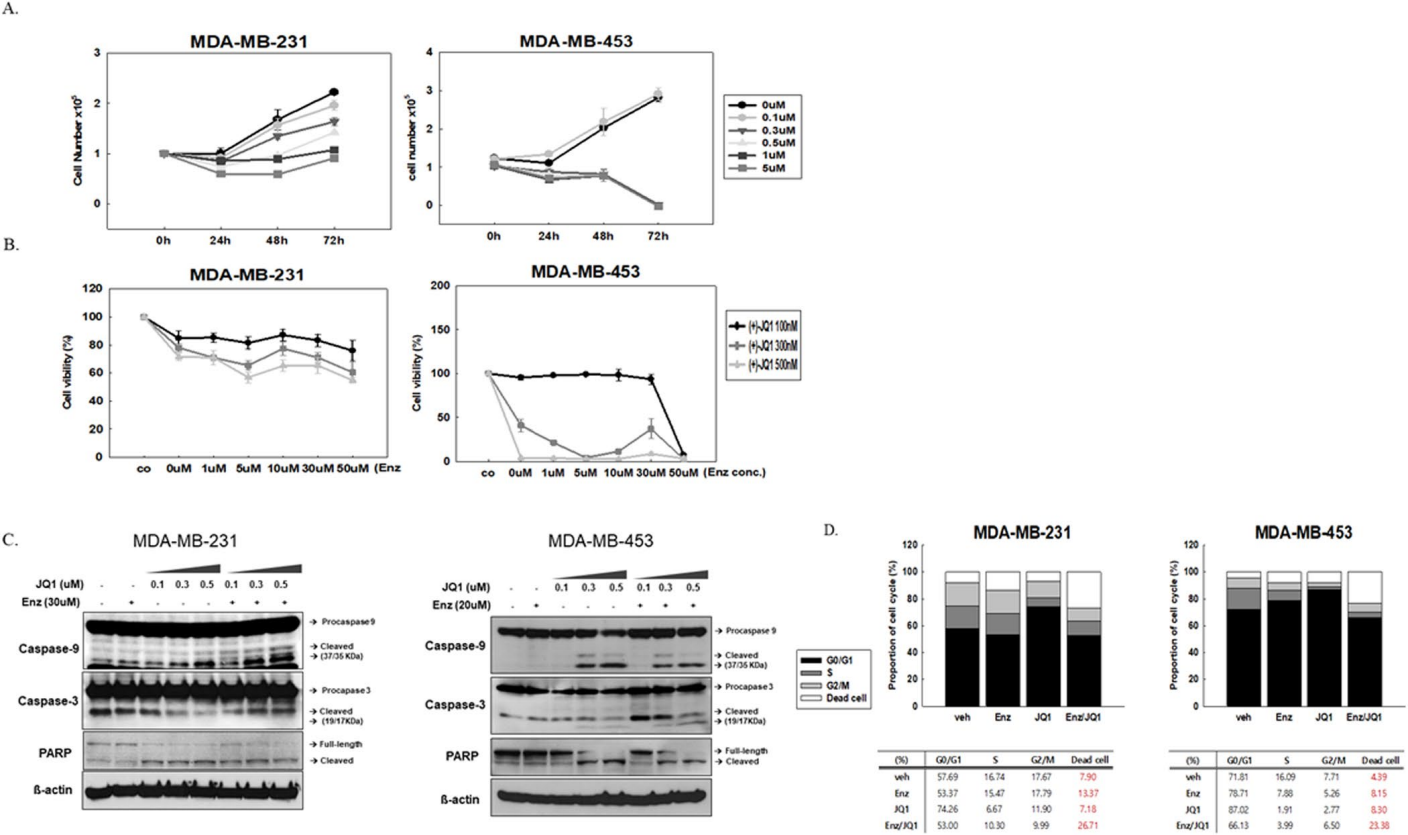
JQ1 exhibits anti-proliferative activity and induces cell cycle arrest. (**A**) Anti-proliferative activity of JQ1 in AR + TNBC cell lines following 72 hours of treatment. (**B**) Cytotoxic assay to test the effect of JQ1/enzalutamide (Enz) combination in AR + TNBC cell lines following 72 hours of treatment. (**C**) JQ1 treatment with or without Enz (30 uM) induced expression of apoptotic proteins in both cell lines. (**D**) Cell cycle analysis indicated cell cycle arrest at G0/G1 induced by JQ1 treatment (0.5 uM) and JQ1/Enz (0.5 uM/30 uM) combination enhanced cell death.

The BET inhibitor JQ1 showed dose-dependent anti-proliferative activity in AR + TNBC cell lines following 72 hours of treatment (Fig. 2A). JQ1 and enzalutamide combination treatment exhibited enhanced cytotoxic effects against AR + TNBC cells (Fig. 2B). Meanwhile, AR negative TNBC cell lines, MDA-MB-468 and BT-20 showed minimal response to JQ1 treatment (Supplemental Fig. 2). The combination of enzalutamide did not increase the cytotoxic effect of JQ1 in both cell lines (Supplemental Fig. 2). In AR + TNBC cell lines, JQ1 treatment induced apoptosis in a dose-responsive manner, as evidenced by increased levels of cleaved forms of caspase-3, caspase-9, and PARP. These effects were enhanced in both cell lines by the addition of enzalutamide (Fig. 2C). We also assessed the effect of JQ1 on AR + TNBC cell cycle distribution. JQ1 treatment increased the accumulation of cells in the G0/G1 stage, indicative of cell cycle arrest. The combination treatment further increased the population of dead cells (Fig. 2D). Taken together, these data indicate that JQ1 treatment exerted an anti-proliferative effect on AR + TNBC cells by inducing apoptosis and blocking cell cycle progression, and this effect was enhanced by enzalutamide-mediated AR blockade.

**Figure 3:**
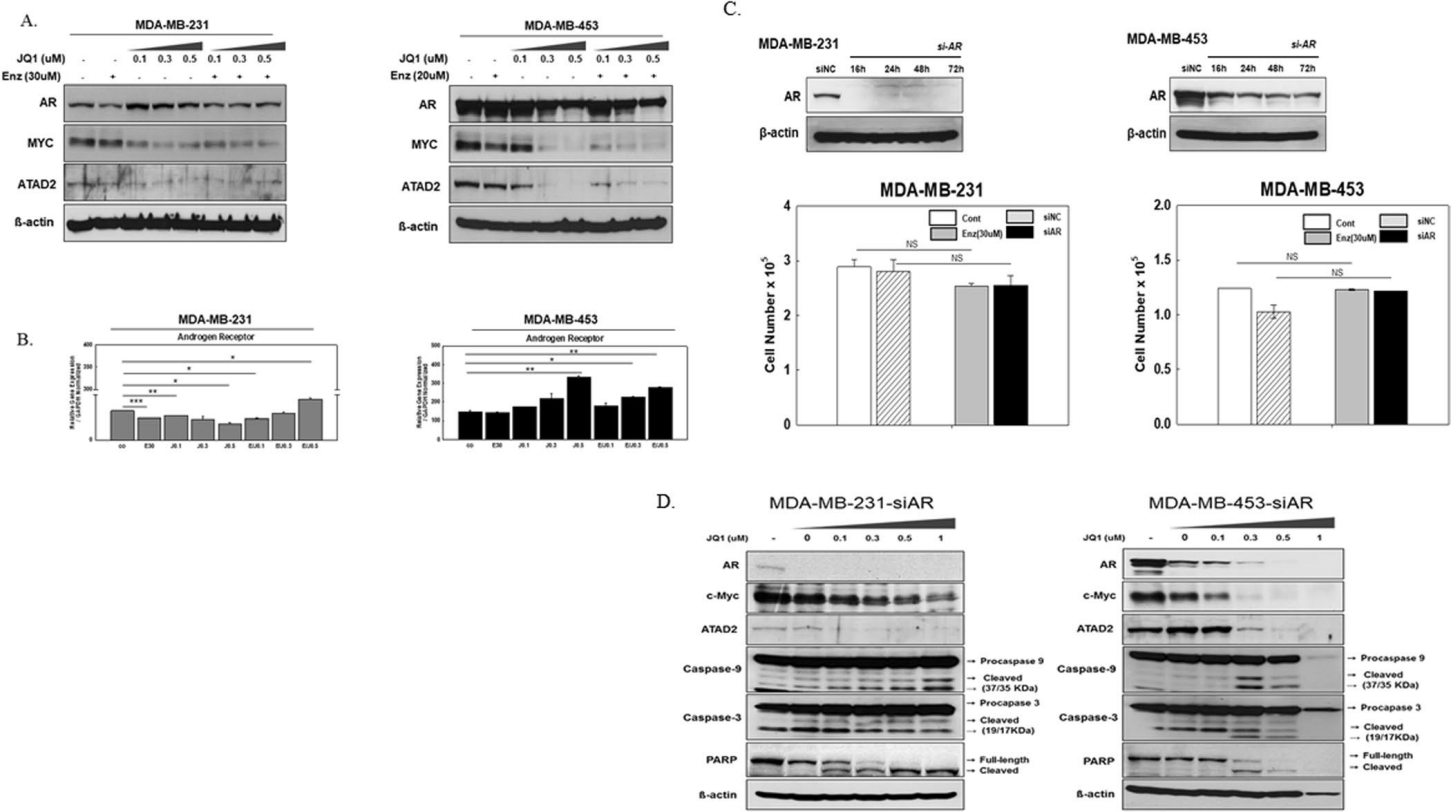
JQ1 suppresses MYC and ATAD2 expression and induces cell apoptosis regardless its effects on AR induction (**A**) The expression of target proteins, MYC, ATAD2, and AR in MDA-MB-231 and MDA-MB-453 cells following 72 hours of JQ1/enzalutamide combination treatment. (**B**) The changes of RNA transcripts of AR according to JQ1/enzalutamide treatment in both cell lines. All experiments were duplicated. (**C**) Introduction of siRNA targeting *AR* (si-AR) in both cell lines. Baseline levels of AR in both cell lines were examined by 72 hours after transfection of *si-AR* (upper). Comparison of the effects on cell viability of AR blockade mediated by *si-AR* and by enzalutamide treatment (bottom). (**D**) The expression of apoptotic proteins, MYC and ATAD2 were assessed following JQ1 treatment of MDA-MB-231 and MDA-MB-453 cells transfected with si-AR. Error bars represent mean ± SE (n = 3) from one of two independent experiments. NS (not significant), *P ≤ 0.01, **P ≤ 0.005, and ***P ≤ 0.001, as determined using a two-tailed Student’s *t*-test.

Next, we sought to define the molecular mechanisms underlying JQ1, BET inhibitor activity in AR + TNBC cells. Considering their previously established link to AR expression in TNBC, we focused on AR, MYC, and ATAD2 as potential targets^21–23^. First, we analyzed the expression of the respective genes in TNBC cells following JQ1 treatment with or without enzalutamide. Following 72 hours, robust reduction in *MYC* and *ATAD2* expression occurred following JQ1 treatment (Fig. 3A). On the other hand, the JQ1 and enzalutamide combination did not completely inhibit AR expression, but rather that the expression of AR increased with higher JQ1 concentration in both cell lines (Fig. 3A,B). To determine whether the induction of AR may offset the effect of JQ1 treatment, we used siRNA to abrogate *AR* expression (*si-AR*). Despite confirmed AR suppression, the *si-AR* produced a cytotoxic effect comparable to that elicited by the enzalutamide treatment alone and did not significantly alter MYC and ATAD2 levels relative to the control siRNA (Fig. 3C,D). The expression of apoptotic proteins such as cleaved forms of caspase-3, caspase-9, and PARP were observed when JQ1 was added, which was comparable to those of JQ1/enzalutamide combination (Fig. 3D).

### JQ1 suppressed expression of AR-associated genes by disrupting BRDs-AR interactions

**Figure 4:**
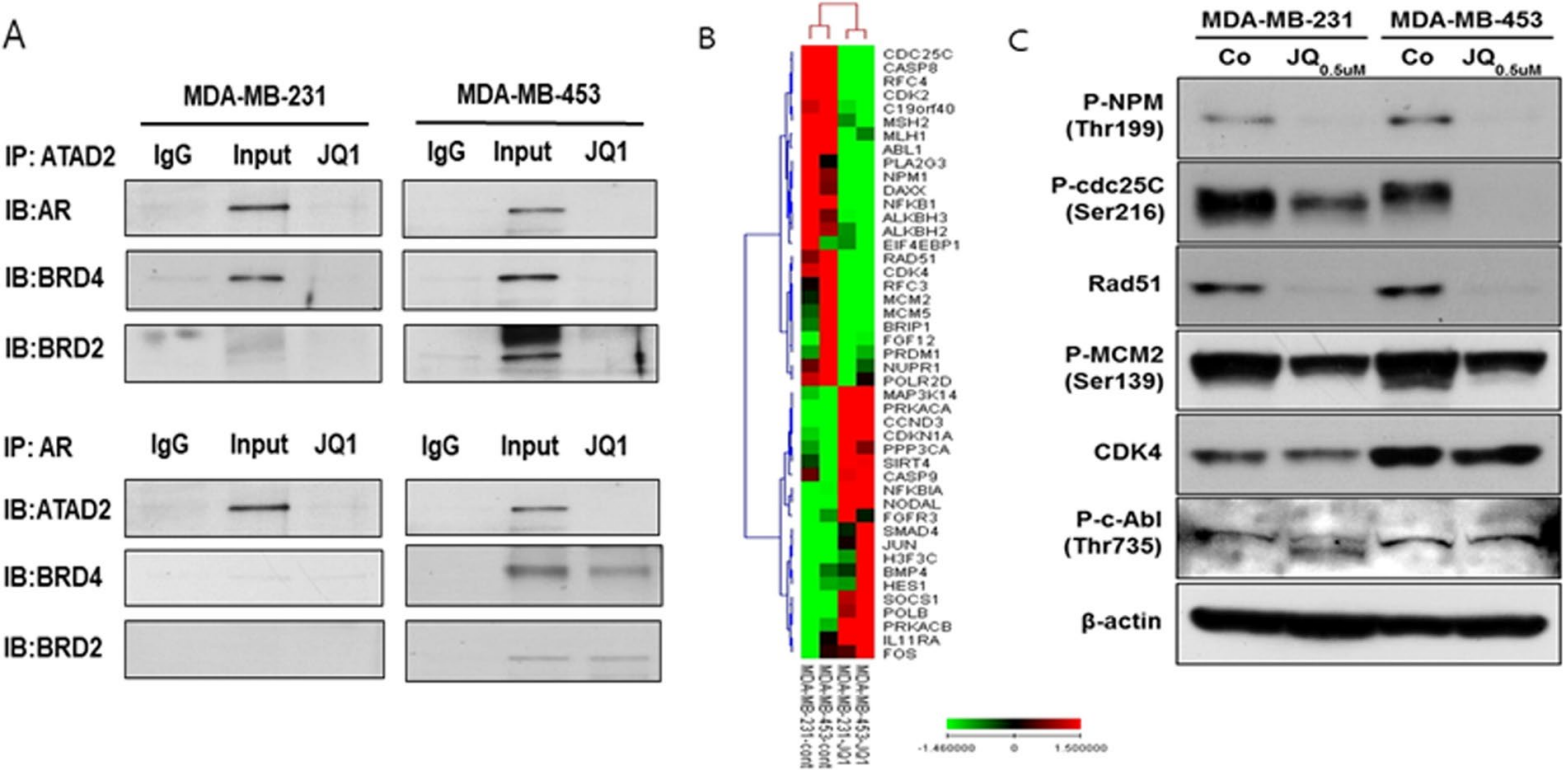
JQ1 treatment disrupts BRDs protein interactions with AR and alters AR-associated gene expression. (**A**) Immunoprecipitation assay to assess the interaction among ATAD2, AR, BRD2, and BRD4. MDA-MB-231 and MDA-MB-453 cells were treated with DMSO (vehicle) or 0.5 μM JQ1 for 72 hours. The nuclear proteins of the cells were isolated, and the interaction between AR or ATAD2 and each protein was assessed via immunoprecipitation. (**B**) Heatmap demonstrating changes in gene expression patterns in response to JQ1 (0.5 uM) treatment for 72 hours. (**C**) Western blotting to examine the effect of JQ1(0.5 uM) treatment on the abundance of indicated proteins. Data shown as mean ± SE (n = 3) from one of two independent experiments. NS, not significant; *P ≤ 0.01, **P ≤ 0.005, and ***P ≤ 0.001, as determined using a two-tailed Student’s *t*-test.

Next, we used siRNAs to suppress *ATAD2*, *BRD2*, *BRD4*, and *MYC* expression to determine which factors were responsible for the observed JQ1 effects. However, knockdown of any of the genes alone did not reduce cell viability relative to treatment with non-targeting siRNA (Supplemental Fig. 2). To further investigate the role of JQ1 in AR expressing breast cancer cells, we assessed protein-protein interactions among ATAD2, AR, as well as JQ1 targets including BRD2, and BRD4. JQ1 treatment did not affect the interactions between AR and BRD2/BRD4 (Fig. 4A). On the other hand, ATAD2 strongly bound to AR and BRD4, and exhibited a weak interaction with BRD2. JQ1 treatment completely interfered with these interactions (Fig. 4A).

To identify JQ1-modulated genes and their role in AR signaling, we performed multiplex gene expression analysis using JQ1-treated AR + TNBC cells. Transcripts downregulated by JQ1 treatment comprised genes predominantly associated with cell cycle regulation and DNA damage response (Fig. 4B, Supplemental Table 1). Most significantly downregulated genes such as phosphor-nucleophosmin (NPM), phospho-M-phase inducer phosphatase 3 (CDC25C), RAD51, and phopho-MCM2 were known to interact with AR for androgen-dependent transcriptional regulation. We subsequently validated decreased protein levels of phospho- NPM, phospho- CDC25C, RAD51, and phopho-MCM2 upon JQ1 treatment (Fig. 4C).

### JQ1 exhibited *in vivo* activity

**Figure 5:**
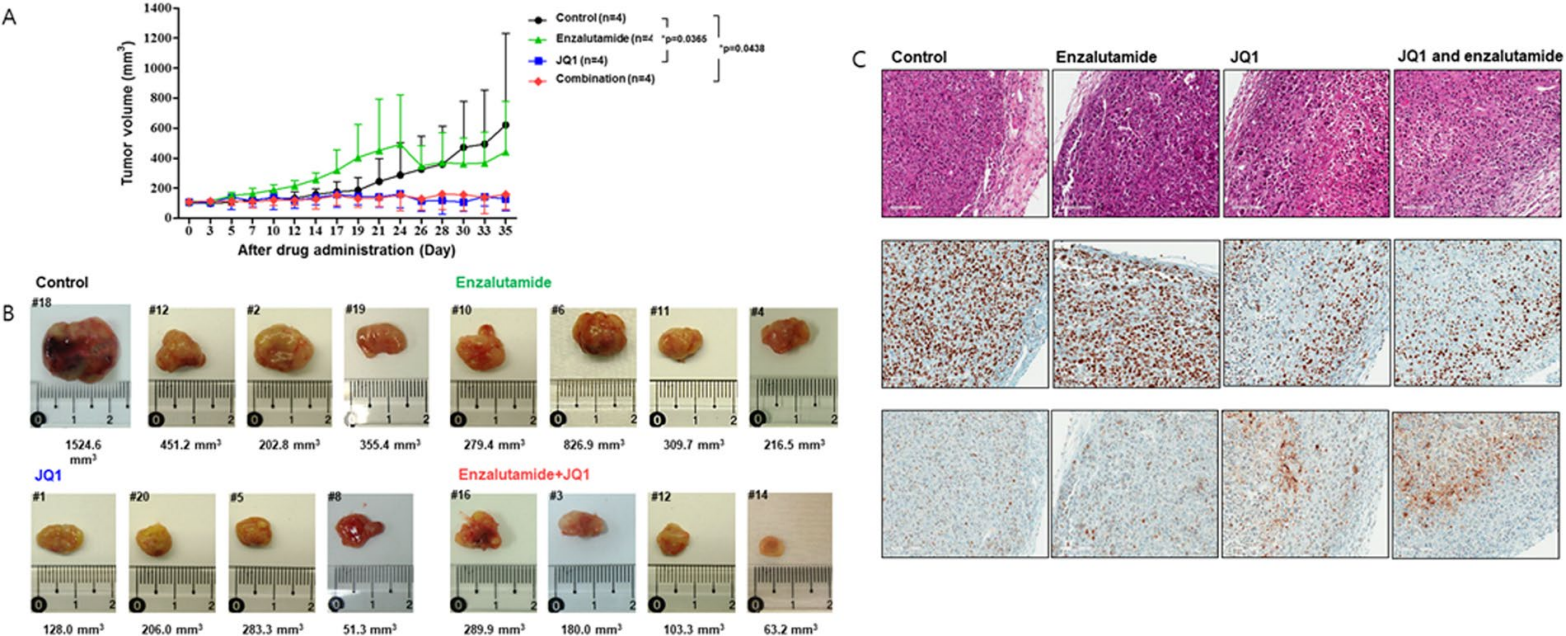
BET inhibitor and AR blockade combination treatment in an *in vivo* xenograft mouse model. MDA-MB-231 cells were injected into BALB/c nude mice. Enzalutamide, JQ1, or combination treatments were administered to the animals (n = 4 per group) for ~3 weeks until the tumor volume reached 100 mm^3^. (**A,B**) Measurement of tumor volumes. (**C**) Hematoxylin and eosin staining (upper), immunohistochemistry staining for KI-67 (middle) and cleaved caspase-3 (bottom). Microscopy images were obtained using 20× magnification. Data shown as mean ± SE (n = 4). NS, not significant; *P ≤ 0.01, as determined using a Mann-Whitney U test.

We next used the MDA-MB-231 xenograft mouse model to investigate the *in vivo* therapeutic effect of JQ1. The JQ1 dose (50 mg/kg, daily) for mouse experiments was selected by referring to the previous papers^24,25^. JQ1 treatment exhibited robust anti-tumor activity (Fig. 5). Manual measurements demonstrated that JQ1 treatment reduced tumor volume on average by 60% (Fig. 5A,B). Combination of enzalutamide and JQ1 resulted in comparable suppression of tumor growth. However, no statistically significant differences were observed in tumor suppression elicited by JQ1 alone. JQ1 with or without enzalutamide combination treatment also significantly reduced Ki-67 levels and increased caspase-3 cleavage in tested tumor sections (Fig. 5C).

## Discussion

Maintenance of AR signaling is one of the most common resistance mechanisms to conventional hormonal treatments in patients with advanced prostate cancer^26^. The LAR subtype of TNBC is associated with increased *AR* gene expression, as well as with elevated nuclear and total AR protein levels. AR expression in LAR subtype tumors is responsible for tumor cell viability and survival, with knockdown of *AR* expression significantly reducing the ability of LAR cell lines to form colonies^3,5^.

Preclinical *in vitro* and xenograft studies have demonstrated that LAR cell lines are sensitive to AR inhibition^3^. Other TNBC subtypes also express *AR* and utilize AR signaling for cancer cell survival^9^. Therefore, AR inhibition represents a potential therapeutic strategy for targeting certain TNBC subtypes. However, AR antagonists, such as bicalutamide and enzalutamide, have demonstrated modest efficacy in clinical trials, with 12 to 14.7 weeks of median progression-free survival^11,27^. In these clinical trials, all patients with AR expression above 1% in their tumor tissue were included and the association between AR expression and the efficacy of AR antagonists have not been assessed yet. Currently, many clinical trials are assessing combination therapies to enhance the efficacy of AR antagonists in breast cancer^28^.

In this study, we observed that the combination of the BET inhibitor, JQ1, and the AR antagonist, enzalutamide, induced cell cycle arrest and apoptosis of TNBC cells. Previous studies found that epigenetic modulation by BET inhibitor showed significant cytotoxic effects in TNBC and overcame the resistance of many cytotoxic chemotherapy^29^. Especially, BET inhibitor, JQ1 displaced BRD4, which caused the downregulation of genes related with cell cycles, proliferation, and invasion^30^. We selected MDA-MB-231 and MDA-MB-453 cell lines which showed low and strong AR expression respectively. While MDA-MB-453 cell line is well known to belong to the LAR subtype and MDA-MB-231 cell line is known as the mesenchymal subtype^3^. Although MDA-MB-231 cell line has a controversial issue if it expresses AR^3,31^, we used low AR- expressing MDA-MB-231 which seems to reflect well the characteristics of patient population enrolled in many clinical trials using anti-AR therapies. AR + TNBC cell lines were more sensitive to JQ1-containing treatments than to enzalutamide alone. JQ1 did not suppress the expression of AR directly. Rather, following 72 hours of treatment, JQ1 induced AR expression. These findings were consistent with a previous study indicating that JQ1 treatment changes luminal marker levels^30^. Cytotoxic effects of JQ1 were still observed even when AR was induced. However, these effects were enhanced when AR was inhibited by enzalutamide or si-AR transfection. Unlike the *in vitro* study, synergistic effects of JQ1 with enzalutamide was not so clear in *in vivo* experiments. There were two reasons to consider. First, the AR expression in graft tumors was low, which diminished the combination effect of enzalutamide. Second, the dose of JQ1 of 50 mg/kg was too high to observe the synergistic effect with other drugs, even though JQ1 alone showed maximal effect at that dose level. We chose that single dose level by referring to the previous studies with expecting rare toxicity and maximum cytotoxic effects^24,25^. The concentration of JQ1 which may show a synergistic effect with enzalutamide, may be lower and a dose finding study should be needed for further development.

In this study, expression of MYC target of JQ1 showed no clear association with JQ1 sensitivity, which was in line with previous study^30^. Instead, JQ1 appeared to exert its cytotoxic effects by inhibiting the interaction of ATAD2 with BRD4 and AR. ATAD2 acts as a transcriptional co-regulator of estrogen receptor alpha and AR to promote the expression of genes driving cancer cell proliferation and survival^22,23^. ATAD2 overexpression is a poor prognosis marker in various cancers, especially in TNBC^21,32,33^. ATAD2 harbors BRD^21^ and acts as a cofactor for MYC. Together with BRD4, ATAD2 acts as a co-activator of AR, maintaining AR signaling in prostate cancer^17,20^. However, its expression was not affected by AR blockade or AR knockdown in our study. In contrast, JQ1 treatment significantly downregulated ATAD2 and BRD4 expression. However, the reductions in ATAD2 or BRD4 levels alone were not sufficient to decrease cell viability and induce cell cycle arrest. Instead, our results indicate that the JQ1 effect on AR + TNBC cells is predominantly driven by disrupted interactions of ATAD2 with BRD2, BRD4, and AR. By interrupting the interaction of AR with other proteins, JQ1 effectively downregulates the levels of AR targets involved in DNA damage response and cell cycle regulation, including AR interacting genes such as phospho-NPM, phospho-CDC25C, RAD51, and phopho-MCM2^34–39^. The importance of AR in cell cycle control has been underscored by previous findings of AR impacting cell cycle regulation even in the absence of the AR ligand^40^. Our findings indicate that JQ1 likely interrupts the crosstalk between AR and the cell cycle machinery, thereby suppressing the effect of AR on cancer cell survival. Urbanucci *et al*., showed that castration-resistant prostate cancer (CRPC) had chromatin accessibility which may drive cancer progression and AR/androgen -regulated BRDs including ATAD2 mediated this effect^20^. They also suggested that ATAD2 and BRD2 overexpression have poor prognostic value. In line with their results in CRPC, our findings indicate that the BET inhibitor JQ1 is more efficacious than direct AR blocade in AR + TNBC cell lines and in xenograft models. These results suggest that clinical evaluation of BET inhibition, either as a monotherapy or in combination with anti-AR agents, is warranted in the exploration of novel approaches for targeting AR + TNBCs.

## Conclusions

The BET inhibitor JQ1 enhances cytotoxic efficacy of therapeutic interventions against of AR + TNBCs. Anti-proliferative effects of JQ1 are likely driven by the disruption of ATAD2 interactions with BRD2, BRD4, and AR which leads to down-regulate AR associated genes. Clinical trials are needed to establish the efficacy and utility of using BET inhibition in combination with anti-AR therapy for the treatment of AR + TNBC.

## Supplementary information

supplementary figures

## Data Availability

All data generated or analysed during this study are included in this article and its supplementary information files.
