# Supplementary material for: Anti-tumor activity of BET inhibitors in androgen-receptor-expressing triple-negative breast cancer
Source: Sci Rep. 2019 Sep 16;9:13305. doi: 10.1038/s41598-019-49366-9 (PMC6746817; doi:10.1038/s41598-019-49366-9)
Supplement: Supplementary file 1 — supplementary figures [file 41598_2019_49366_MOESM1_ESM.pdf]

Anti-tumor activity of BET inhibitors in androgen-receptor-expressing triple-negative breast cancer

Running title : BET inhibitor for AR positive TNBC

In Hae Park[1,2*], Han Na Yang[1], Su Yeon Jeon[1], Jung-Ah Hwang[3], Min Kyeong Kim[1], Sun-Young Kong[1,4,5], Sung Hoon Shim[1,2], Keun Seok Lee[2]

1. Translational Cancer Research Branch, Division of Cancer Research, National Cancer Center, Goyang, Korea
2. Center for Breast Cancer, National Cancer Center, Goyang, Korea
3. Genomic core, Omics Core Laboratory, National Cancer Center, Goyang, Korea
4. Cancer Biomedical Science, National Cancer Center Graduate School of Cancer science and Policy, Goyang, Korea
5. Department of Laboratory Medicine, Center for Diagnostic Oncology, National Cancer Center, Goyang, Korea

***Corresponding author**

In Hae Park, MD., PhD., E-mail: parkih@ncc.re.kr. Center for Breast Cancer, Translational Cancer Research Branch, Division of Cancer Research, National Cancer Center, Goyang, Korea. 323 Ilsanro Ilsandonggu Goayngsi Geyunggido Korea. Tel : +82-31-920-1680.

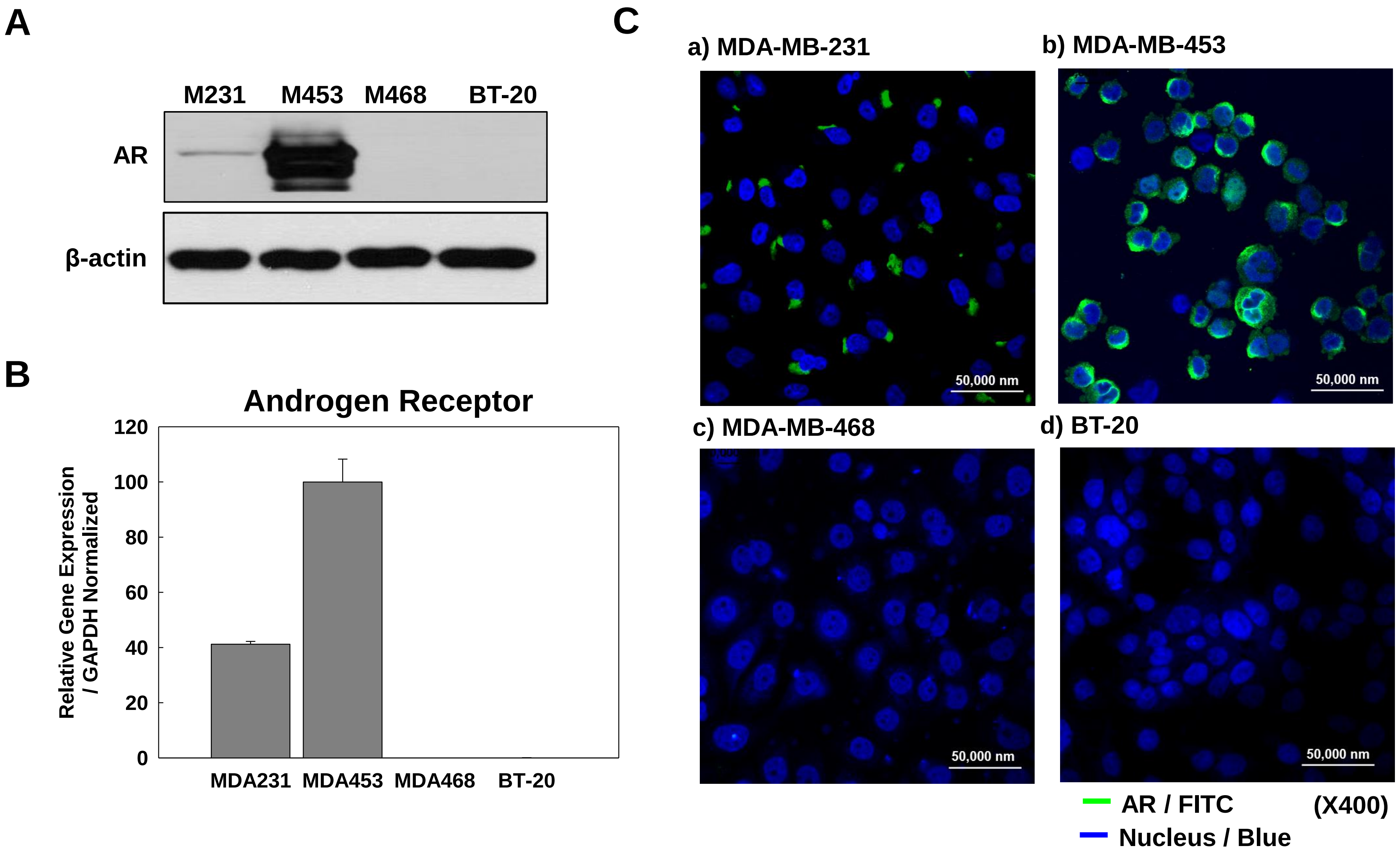

Supplemental Fig. 1. Expression patterns of androgen receptor (AR) in TNBC cell lines. (A) Western blot analysis of AR protein levels in indicated cell lines. (B) Relative *AR* mRNA levels as determined by qPCR. (C) Detection of AR in each cell using immunohistochemistry. AR and nuclei were labeled with FITC (green) and DAPI (blue), respectively. Error bars represented standard error of the mean (SEM).

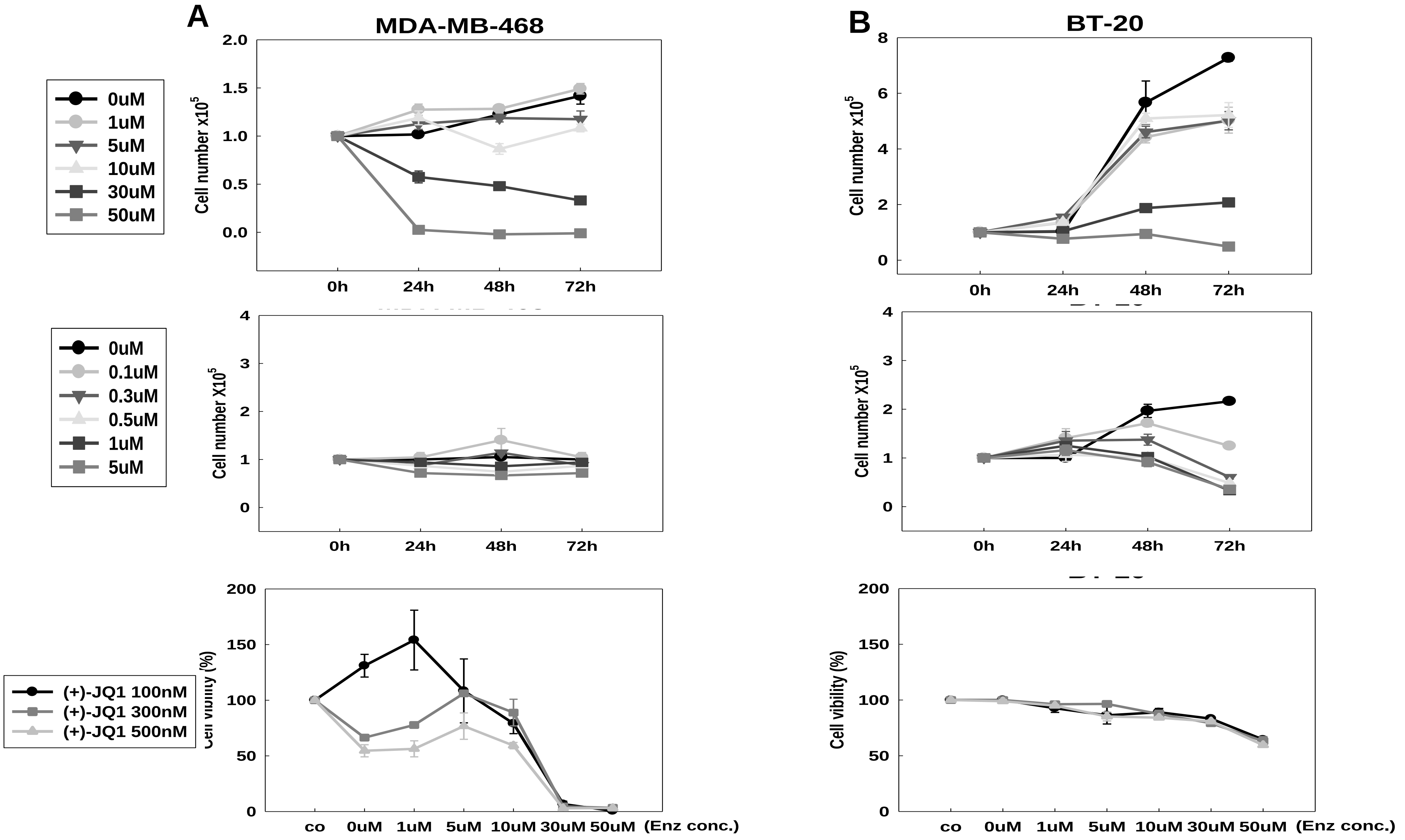

Supplemental Fig. 2. Cytotoxic assay with enzalutamide, JQ1 and combination (A) MDA-MB-468 and (B) BT-20 cell lines. Enzalutamide (upper), JQ1 (middle), and combination (lower).

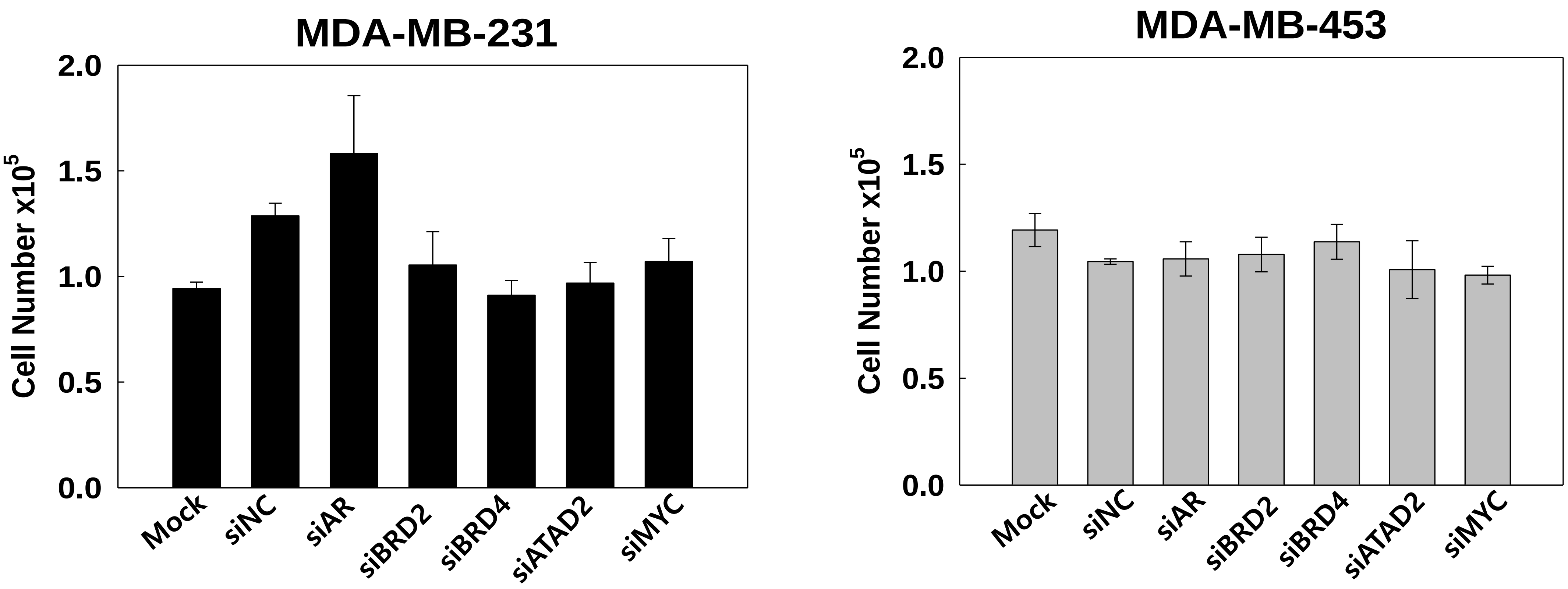

Supplemental Fig. 3. The effects of ATAD2, BRD2, BRD4, and MYC abrogation by siRNAs on cell viability. Error bars represent mean±SE (n=3)
